# Mannoheptulose has differential effects on fasting and postprandial energy expenditure and respiratory quotient in adult Beagle dogs fed diets of different macronutrient contents

**DOI:** 10.1017/jns.2014.17

**Published:** 2014-08-13

**Authors:** Leslie L. McKnight, Elizabeth A. Flickinger, James France, Gary M. Davenport, Anna K. Shoveller

**Affiliations:** 1 Centre for Nutrition Modelling, Department of Animal and Poultry Science, University of Guelph, 50 Stone Road East, Guelph, Ontario, Canada N1G 2W1; 2 Procter and Gamble (P&G) Pet Care, 8700 Mason-Montgomery Road, Mason, OH, 45040, USA

**Keywords:** Mannoheptulose, Beagles, Energy expenditure, Respiratory quotient, CHO, carbohydrate, EE, energy expenditure, ER, energy restriction, HC, high-carbohydrate (low-fat) diet with placebo supplement, HC, high-carbohydrate (low-fat) diet with mannoheptulose (8 mg/kg)-containing supplement, LC, low-carbohydrate (high-fat) diet with placebo supplement, LC, low-carbohydrate (high-fat) diet with mannoheptulose (8 mg/kg)-containing supplement, MH, mannoheptulose, RQ, respiratory quotient, TEF, thermic effect of food

## Abstract

The present study aimed to determine the effects of mannoheptulose (MH) (8 mg/kg) on energy expenditure (EE), respiratory quotient (RQ) and glycaemic response in healthy adult Beagle dogs (*n* 8; 9·62 (sem 0·31) kg; body condition score 4·5). The study was designed as replicated 4 × 4 Latin squares with a 2 × 2 factorial treatment structure. The dietary treatments were low carbohydrate (CHO) relative to fat diet (LC; 31 % CHO, 28 % fat) with placebo (0 mg/kg) or MH supplement and high CHO relative to fat diet (HC; 54 % CHO, 11 % fat) with placebo (0 mg/kg) or MH supplement. Dogs were fed to maintain body weight (HC and HC^+MH^ 3625 (sem 295) kJ and LC and LC^+MH^ 3542 (sem 284) kJ). Resting and postprandial (0–4 h; 5–10 h; 11–17 h; 18–23 h) EE and RQ were determined by indirect calorimetry (days 12 or 14). Glycaemic response to a meal (24 h) and plasma MH concentrations were determined on days 12 or 14. Plasma MH followed first-order kinetics, confirming that MH is absorbed and available to the animal. In the presence of high dietary CHO, MH increased postprandial EE (5–10 h only), suggesting MH increased dietary induced thermogenesis. In contrast to earlier reports, MH did not affect serum glucose or insulin in the present study. Irrespective of MH, dogs adapted RQ to diet composition and dogs consuming the LC diet had a greater incremental AUC for glucose, but not insulin, than dogs consuming the HC diet.

Obesity is a major health concern for companion animals, with 55 % of dogs in the USA reported to be overweight or obese^(^^1^^)^. As in humans, canine obesity is associated with metabolic diseases (i.e. diabetes), and the aetiology is multi-factorial (i.e. genetic disposition, advancing age and sedentary lifestyle). Overweight dogs are commonly treated with nutritional management strategies that include specially formulated therapeutic (weight-loss) diets and/or total energy restriction (ER)^(^^2^^)^. Weight-loss diets achieve energy dilution by altering the macronutrient content of the diet. Generally these diets contain low fat and high carbohydrate (CHO) concentrations. As dogs preferentially utilise fat over CHO for energy^(^^3^^)^, it is unclear whether increased inclusion of dietary CHO relative to fat incurs any metabolic benefit beyond energy dilution for managing obesity. Studies in human subjects have been controversial, with some reporting greater weight loss in individuals consuming high-fat, low-CHO diets^(^^4^^,^^5^^)^. It is well established that ER, without malnutrition, is the most robust and repeatable strategy for weight management. In addition, ER has been shown to delay the ageing process by increasing median lifespan in yeast, flies, nematodes, rodents and dogs^(^^6^^,^^7^^)^. Studies in human subjects and non-human primates have not found ER to increase lifespan, but have shown that ER provides protection from age-related disease development^(^^8^^,^^9^^)^ that would result in increased quality of life. How ER exerts these beneficial effects is incompletely understood. However, increased metabolic efficiency, enhanced insulin-signalling pathways, and reduced oxidative damage are all hallmarks of ER^(^^9^^)^. Unfortunately, ER generally results in significant behavioural changes in pets that are often perceived as negative by the owner (i.e. begging and aggression), thereby straining the human–animal bond^(^^10^^)^.

ER mimetics provide an alternative to ER. As the name implies, compounds with ER mimetic activity mimic the metabolic, hormonal and physiological effects associated with ER without altering dietary energy intake^(^^11^^)^. Mannoheptulose (MH) is a seven-carbon sugar found in avocados that has been proposed as an ER mimetic^(^^12^^)^. Early research demonstrated that MH functions as a glycolytic inhibitor by its ability to competitively inhibit hexokinases (*EC* 2·7·1·1)^(^^13^^)^. Dogs given MH doses ranging 1–2 g/kg (intravenous or intra-arterial) exhibit a transient diabetic state, characterised by marked hyperglycaemia, dramatically decreased insulin:glucagon ratio and increased hepatic glucose output^(^^14^^–^^17^^)^. These responses were well outside normal physiological ranges, suggesting that the dosage was supraphysiological. However, the effects of low doses of MH are largely unknown. Furthermore, early studies only examined the effects of MH on fasting metabolism. As MH inhibits glucokinase in the intestine, liver and pancreas, it is of interest to examine the effects of MH on postprandial metabolism.

The objective of the present study was to determine the effects of avocado-derived MH on glycaemic response, macronutrient oxidation and energy expenditure (EE) in adult Beagle dogs. Given MH's known role as a glycolytic inhibitor, we expected MH to differentially affect these outcomes when fed in the presence of low or high dietary CHO concentrations.

## Experimental methods

### Animals and housing

All procedures were approved by the Institutional Animal Care and Use Committee of Procter and Gamble (P&G) Pet Care (Mason, OH, USA). A total of eight adult Beagle dogs (seven spayed females and one neutered male, 9·62 (sem 0·31) kg body weight; 7·55 (sem 0·39) years of age) were used in the present study. All dogs were at optimal body condition score 4·5 (nine-point scoring scale^(^^18^^)^) at the initiation of the study. All dogs resided at the P&G Pet Health and Nutrition Center (Lewisburg, OH, USA) and were considered healthy based on a general health evaluation by a licensed veterinarian before the study. Dogs were pair-housed with free access to water and indoor and outdoor runs. Indoor runs were maintained on a 12 h light and dark cycle, in addition to natural light, and were equipped with raised canvas beds, toys and heated flooring. Outdoor runs were equipped with toys and play yard equipment. All dogs received 40 min of supervised group exercise and socialisation in a separate fenced yard daily.

### Study design

The study was designed as replicated 4 × 4 Latin squares with a 2 × 2 factorial treatment (diet) structure. Each dog (*n* 8) was randomly allocated to a Latin square sequence defining the order to receive the four dietary treatment combinations. The total duration of the study was 56 d with each treatment leg (or period) lasting 14 d. Indirect calorimetry and a meal challenge were performed during each 14-d treatment leg. The indirect calorimetry method only allowed for four dogs to be measured per d (four calorimetry chambers total). Dogs were divided into two groups of four balanced by treatment sequence (each diet × MH combination represented on each day). Indirect calorimetry was performed on day 12 for group 1 followed by the meal challenge on day 14. In contrast, dogs in group 2 underwent the meal challenge on day 12 followed by indirect calorimetry on day 14.

### Diets and feeding

The dietary treatments were a low CHO relative to fat diet with a placebo supplement (LC) or MH-containing supplement (LC^+MH^) and a high CHO relative to fat diet with a placebo supplement (HC) or MH-containing supplement (HC^+MH^). The diets were nutritionally balanced and complete and made of identical ingredients and formulated to deliver an equivalent protein:energy ratio (Table 1). The supplements were made of cocoa butter with a baker's white coating (94 % DM, 25 % fat, 5·7 % protein, 5·9 % ash, 0·2 % crude fibre). MH was incorporated into the supplement by mixing whole-fruit avocado extract with the cocoa butter and baker's white coating. The MH-enriched avocado extract was produced using commercially available avocados (Hass variety). Frozen, whole avocados comprised of the flesh, peel and pit were initially ground before suspension in water (1:3, w/w). The resultant slurry was centrifuged to remove non-aqueous solids. A series of microfiltration (de-oiling), ultrafiltration (10 kDa) and nanofiltration (100 kDa) was used to produce the MH-enriched fraction. Lyophilisation was used to form the final crystalline powder yielding 18 % MH^(^^19^^)^.

**Table 1.** Ingredient and chemical composition of the test diets, formulated with low (LC) and high (HC) concentrations of dietary carbohydrate

Composition	LC	HC
Ingredient (%)		
Maize meal	18·5	32·0
Sorghum grain, ground whole	18·5	31·9
Chicken meal	18·5	12·8
Chicken byproduct meal	18·4	12·8
Chicken fat*	15·6	2·0
Beet pulp, dried	3·6	2·9
Chicken broth	2·0	1·9
Chicken liver	1·0	1·0
Fish oil*	0·7	0·4
Mineral mix†	2·1	1·7
Vitamin mix‡	0·4	0·3
Analysed chemical profile (DM basis)		
DM (%)	92·8	90·8
Crude protein (%)	32·2	27·0
Fat (%)	27·9	11·2
Crude fibre (%)	2·2	2·6
Ash (%)	7·1	5·6
NFE (%)§	30·6	53·6
Gross energy (kJ/g)	24·5	20·9
Metabolisable energy (kJ/kg)‖	19 131	24 672
Protein:gross energy ratio	1·3	1·3
Mannoheptulose (ppm)	0	0

NFE, N-free extract; ppm, parts per million.

* Preserved with mixed tocopherols.

† Sodium chloride, calcium carbonate, potassium chloride, monosodium glutamate, ferrous sulfate, zinc oxide, manganese sulfate, copper sulfate, manganous oxide, potassium iodide, cobalt carbonate.

‡ Vitamin E, choline chloride, ascorbic acid, vitamin A acetate, calcium pantothenate, biotin, thiamin mononitrate, vitamin B_12_, niacin, riboflavin, inositol, pyridoxine hydrochloride, vitamin D_3_, folic acid.

§ NFE was calculated as follows: % NFE = 100 % – (% moisture + % crude protein + % ash + % crude fat + % crude fibre).

|| Metabolisable energy content was determined using the modified Atwater calculation, where fat, protein and carbohydrate provide 35·6, 14·7 and 14·7 kJ/g (8·5, 3·5 and 3·5 kcal/g), respectively.

Dogs were fed to maintain body weight, based on their historical energy intake records (HC and HC^+MH^ 3625 (sem 295) kJ and LC and LC^+MH^ 3542 (sem 284) kJ). For 1 week before study initiation dogs were fed their initial test diet (‘wash-in’ period). Dogs were individually fed their daily ration of test diet in two meals (08·00 hours and 13·00 hours). At each meal, dogs received a supplement containing either 0 mg MH (placebo) or 40 mg MH. The daily dose of MH was about 8 mg/kg for each dog (two supplements per dog per d).

### Indirect calorimetry

Respiratory gas exchange measurements were conducted via whole-body indirect calorimetry on day 12 or 14 of each treatment leg. The calorimetry chambers (76 cm × 53 cm × 61 cm, length × width × height) were made of clear Plexiglas and fitted with a hinged access top door for providing food to the dog. Chambers were designed as open-circuits with room air pulled into the chambers at a rate of 9–18 litres/min to maintain CO_2_ levels in the chamber between 0·4 and 0·8 %. Exiting chamber air was dried by passing it through columns of Drierite™ and magnesium perchlorate before reaching the O_2_ and CO_2_ analysers (Qubit Systems Inc.). Each chamber was sampled every 3 s over a 5 min period. O_2_ and CO_2_ exchange and respiratory quotient (RQ) data were logged using data acquisition software (Qubit C950-MCGES; Qubit Systems Inc.). Before study initiation, dogs were acclimatised over an 8-week period (minimum exposure of 1 h per week) to rest comfortably in the chamber with no excessive activity or movement and were discouraged from urinating or defecating in the chamber.

Gas exchange measurements consisted of two fasting collections occurring 18 h after the dog's last meal (time –50 min and –25 min). Dogs were then fed their full daily ration of test diet and supplements as a single meal (considered time zero). After feeding, gas exchange measurements were collected every 25 min for 23 h. Dogs were provided a brief 10-min break at 4, 10 and 17 h post-feeding in which they were removed from the chamber, taken outdoors into a fenced area and provided an opportunity to urinate and defecate. During these breaks the O_2_ and CO_2_ analysers were re-calibrated. After each break, dogs rested in the calorimetry chambers for a minimum of 25 min to ensure adequate CO_2_ accumulation before the resumption of gas exchange measurements. EE was calculated from O_2_ consumption and CO_2_ production using the abbreviated Weir equation^(^^20^^)^ and expressed on a per kg metabolic body-weight basis (BW^0·75^).

### Glycaemic meal challenge

An eighteen-gauge catheter was inserted into the jugular vein for blood sampling. One fasting blood sample (about 18 h since the last meal) (time –5 min) was collected, after which dogs were fed their full daily ration of test diet and supplements as a single meal (considered time zero). Blood samples were collected at 15, 30 and 45 min, and at 1, 2, 3, 4, 6, 8, 10, 12, 14, 16, 18, 20, 22 and 24 h post-feeding. Blood was centrifuged for 8 min at 3000 ***g*** at 7°C and the serum and plasma were stored separately at –80°C for subsequent analysis of serum glucose and insulin, and plasma MH. The glucose:insulin ratio was calculated at each time point. MH followed first-order elimination kinetics, and the elimination rate constant (*K*) was calculated as the slope of the line from the semi-log plot of MH plasma concentration *v.* time. The half-life (t_1/2_) of MH was calculated as ln(2)/*K* while MH turnover time was calculated as 1·44 × (t_1/2_).

### Biochemical analyses

Serum glucose was measured by a clinical chemistry analyser (Beckman Coulter AU480). Insulin was assayed using a paramagnetic particle, chemiluminescent immunoassay (Beckman). The assay was not canine specific but was validated using canine serum (lower limit of detection 0·03 µIU/ml, upper limit 300 µIU/ml, where 1 µIU/ml insulin = 6·945 pmol/l). HPLC tandem MS (MS/MS) was utilised to determine plasma MH concentrations. Briefly, 200 µl of plasma were diluted with 800 µl MeCN (methyl cyanide) and 10 µl of internal standard solution (^13^C_7_ D-MH; Toronto Research Chemicals, Inc.) and sonicated for 5 min, followed by centrifugation for 5 min at 3750 rcf (relative centrifugal force). The supernatant fraction (10 µl) was injected into a Shodex NH2P-40 3E column (Showa Denko America Inc.), with guard on a Shimadzu LC10AVP HPLC (flow rate 300 µl/min, column temperature 50°C). Mobile phase A was 61 % acetonitrile, 39 % water with 10 mmol/l formic acid. Mobile phase B was 50 % acetonitrile, 50 % water with 10 mmol/l formic acid. The gradient was 100 % A isocratic for 43 min followed by a 6-min gradient to 100 % B. After a 6-min hold at 100 % B, the system was equilibrated at 100 % A for 12 min. MS/MS was performed in negative ionisation mode on a Sciex API 4000 MS/MS. For MH, the formic acid loss was monitored at *m/z* 255 to 209. For C_7_ MH, the formic acid loss was monitored at *m/z* 262 to 216.

### Statistical methods

All data were analysed using the SAS statistical software package (version 9·2; SAS Institute, Inc.) and are expressed as means and pooled standard errors. Mixed-effects models were fitted assuming fixed treatment, period and time effects and dogs as a random variable. Repeated measures within period on dog were analysed using the autoregressive order 1 covariance structure. Multiple comparisons were performed using the Tukey–Kramer method. Results were considered statistically significant if *P* < 0·05. For calorimetry measurements data were analysed in time blocks of 0–4 h, 5–10 h, 11–17 h and 18–23 h postprandial. This analytical approach was taken because the dataset in its entirety cannot be considered continuous, despite the care taken to ensure that all dogs were treated the same during the breaks in collections.

## Results

### Energy expenditure

There were no differences in body weight between dietary treatments or study periods (*P* = 0·99). Resting EE was not affected by diet (Table 2). Postprandial EE was not affected by diet, with the exception of the 5–10 h postprandial period (Table 2, online Supplementary Fig. 1(a)). During this time, dogs that received the HC^+MH^ diet had higher EE than dogs fed the HC (*P* = 0·06) and LC (*P* = 0·04) diets.

**Table 2.** Resting* and postprandial† energy expenditure (EE; kJ/kg^0·75^ per d) and respiratory quotient (RQ) as measured by indirect calorimetry in adult Beagle dogs (*n* 8 in a cross-over design) (Mean values and pooled standard errors)

	HC	HC^+MH^	LC	LC^+MH^	sem	*P*
EE						
Resting	342·0	366·5	387·8	360·8	35·7	0·70
Postprandial, 0–3·8 h	426·4	437·5	457·2	464·7	23·4	0·25
Postprandial, 5–9·6 h	446·7^a^	480·9^b^	443·3^a^	464·0^a,b^	17·0	0·02
Postprandial, 10·9–16·7 h	351·6	366·1	369·4	369·3	14·3	0·71
Postprandial, 18–22·5 h	334·6	335·9	353·1	339·1	13·3	0·28
RQ						
Resting	0·780	0·803	0·764	0·759	0·015	0·10
Postprandial, 0–3·8 h	0·838^a,b^	0·851^a^	0·819^c^	0·824^b,c^	0·007	<0·01
Postprandial, 5–9·6 h	0·914^a^	0·915^a^	0·834^b^	0·828^b^	0·010	<0·01
Postprandial, 10·9–16·7 h	0·918^a^	0·920^a^	0·829^b^	0·825^b^	0·014	<0·01
Postprandial, 18–22·5 h	0·869^a^	0·880^a^	0·797^b^	0·793^b^	0·016	<0·01

HC, high-carbohydrate (low-fat) diet with placebo supplement; HC^+MH^, high-carbohydrate (low-fat) diet with mannoheptulose (8 mg/kg)-containing supplement; LC, low-carbohydrate (high-fat) diet with placebo supplement; LC^+MH^, low-carbohydrate (high-fat) diet with mannoheptulose (8 mg/kg)-containing supplement.

^a,b,c^ Mean values within a row with unlike superscript letters were significantly different (*P* < 0·05).

* Resting measures were taken after an overnight fast (about 18 h since the last meal).

† Postprandial measures were taken for 22·5 h after ingestion of a single test meal and supplements at time zero.

### Macronutrient oxidation

While fasting RQ was not statistically significantly affected by diet, it was highest in dogs fed the HC^+MH^ diet (Table 2). Diet was significant for the entire postprandial period (Table 2, online Supplementary Fig. 1(b)), with dogs being fed the HC and HC^+MH^ diets having significantly higher RQ than dogs fed the LC and LC^+MH^ diets.

### Glycaemic response

**Fig. 1.**
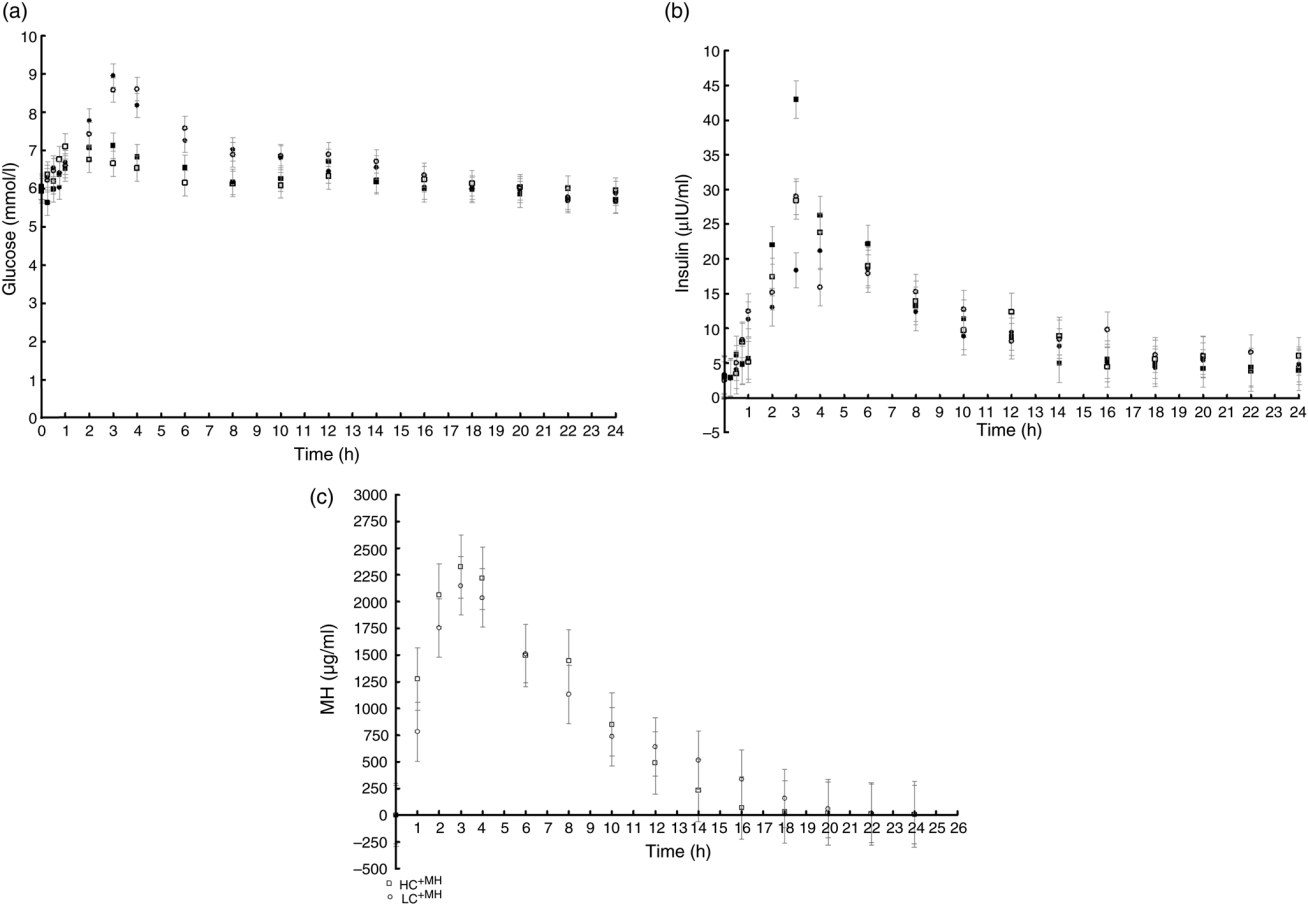
24 h Postprandial serum glucose (mmol/l) (a), serum insulin (µIU/ml, where 1 µIU/ml insulin = 6·945 pmol/l) (b) and plasma mannoheptulose (MH) (µg/ml) (c) in adult Beagle dogs fed their full daily ration of test diet and supplements (low-carbohydrate diet with placebo supplement (LC; ●) or MH-containing supplement (LC^+MH^; ○) and high-carbohydrate diet with placebo supplement (HC; ■) or MH-containing supplement (HC^+MH^; □)) at time zero. Data are means (*n* 8), with pooled standard errors represented by vertical bars, in a complete cross-over design. The main effect of diet was not significant for glucose, insulin or MH.

The blood sampling catheters failed during collections in two dogs (one HC^+MH^ and one HC dog). These dogs were not included in the final analysis. Neither treatment nor period significantly affected serum glucose (Fig. 1(a)), insulin (Fig. 1(b)) or the glucose:insulin ratio (data not shown). However, the incremental AUC for glucose was significantly affected by diet (Table 3). Dogs that received the LC diet had higher incremental glucose AUC than dogs fed the HC diet.

**Table 3.** Incremental AUC (iAUC) for glucose and insulin after ingestion of a single test meal and supplements in adult Beagle dogs (*n* 8 in a cross-over design) (Mean values and pooled standard errors)

	HC	HC^+MH^	LC	LC^+MH^	sem	*P*
iAUC_glucose_	25·6^a^	19·9^a^	36·4^b^	32·0^b^	4·5	0·03
iAUC_insulin_	218	238	202	225	21·5	0·66

HC, high-carbohydrate (low-fat) diet with placebo supplement; HC^+MH^, high-carbohydrate (low-fat) diet with mannoheptulose (8 mg/kg)-containing supplement; LC, low-carbohydrate (high-fat) diet with placebo supplement; LC^+MH^, low-carbohydrate (high-fat) diet with mannoheptulose (8 mg/kg)-containing supplement.

^a,b^ Mean values within a row with unlike superscript letters were significantly different (*P* < 0·05).

### Mannoheptulose kinetics

Analysis revealed no detectable MH in the placebo supplement. Plasma MH followed first-order elimination kinetics in all dogs but one (Fig. 1(c)). Peak MH (2·7 µg/ml) occurred 3·0 h post-feeding, while MH half-life and turnover time averaged 3·7 h and 5·3 h, respectively. MH had returned to non-detectable levels by 24 h.

## Discussion

The present study found that in the presence of high dietary CHO relative to fat, MH increased fasting RQ and postprandial EE (5–10 h). In contrast to earlier reports, MH did not affect serum glucose or insulin in the present study. These findings suggest that MH elicits changes in energy-sensing pathways and macronutrient fuel selection. Interestingly, the present study found that acute consumption (14 d) of a diet low in CHO relative to fat content increased the incremental AUC for glucose (irrespective of MH). This finding persisted despite the dogs' ability to adapt macronutrient oxidation to diet composition.

MH appeared in plasma within 1 h of administration, peaked at 3 h and returned to non-detectable levels by 24 h. Similarly, Davenport *et al.*^(^^21^^)^ observed peak MH concentrations within 2–4 h after oral administration of MH (2 mg/kg) to Labrador retrievers. These findings confirm that MH is absorbed and available to the animal.

While resting EE was not different between diets, it was similar to values reported in dogs in the literature (311–395 kJ/kg^0·75^ per d^(^^22^^)^; 361–370 kJ/kg^0·75^ per d^(^^23^^)^). In the present study, dogs were fed equivalent amounts of dietary protein to energy, and the daily food allowance was set to maintain a dog's body weight over the course of the study. As such, dietary macronutrient differences were not expected to significantly alter whole-body EE. This finding agrees with other studies conducted using dogs^(^^23^^)^ and human subjects^(^^4^^)^ that were fed different concentrations of CHO and/or fat. These observations suggest that energy metabolism is highly controlled in mammals and is not influenced by acute changes in dietary macronutrient concentrations. In contrast, MH increased postprandial EE in the 5–10 h post-feeding period, indicating its ability to affect energy metabolism. It is possible that MH feeding may have increased the thermic effect of food (TEF) based on increased EE. In dogs^(^^24^^)^ and human subjects^(^^25^^)^, increased TEF has been observed for as long as 6 h after a meal. TEF represents the acute increase in EE in response to feeding and accounts for approximately 10 % of daily EE^(^^26^^)^. It can be further divided into obligatory and facultative EE. Obligatory EE is relatively fixed and encompasses energy needs for digestion and absorption. Facultative EE is mediated by sympathetic nervous system activation and β-adrenergic (β-A) responses and can be highly variable^(^^24^^)^. Direct stimulation of the β-A system has been shown to increase postprandial TEF in human subjects^(^^27^^)^. MH can potently stimulate glucagon secretion and inhibit insulin secretion, even in the presence of exogenous insulin infusion^(^^14^^)^. In addition, MH significantly increases hepatic glucose output and circulating cAMP concentrations^(^^15^^)^. All of these metabolic responses are characteristic of a β-A response and may provide a possible mechanism by which MH affects whole-body EE. It should be noted, however, that these previous MH-induced responses resulted from supra-physiological doses (about 1·2 g/kg) of MH administered intra-arterially. In contrast, the observed effects of MH on EE in the present study were associated with a significantly lower level of avocado-derived MH (about 8 mg/kg) administered orally with the diet. Increasing TEF contributes little to daily EE (3–5 %). However, even this small increase in daily EE would be beneficial to overweight and obese animals. The fact that MH increased TEF within the high CHO relative to fat (HC) diet is especially important given that overweight and obese animals are often prescribed therapeutic diets of a similar macronutrient profile. Additional research is warranted to more fully delineate the metabolic mechanism(s) by which MH exerts its effects on whole-body energy metabolism.

Not surprisingly, dogs in the present study demonstrated their ability to adapt macronutrient oxidation to diet composition, as evidenced by changes in the postprandial RQ based on the inclusion of dietary CHO relative to fat. Increased RQ was observed in the HC-fed dogs, suggesting an increased proportion of CHO to fat oxidation. Conversely, a lower RQ indicates a greater proportion of fat relative to CHO oxidised, as seen in dogs consuming the low CHO relative to fat (LC) diet. It is important to note that RQ does not indicate net CHO and fat oxidation, nor does it account for protein oxidation. Only one other study to our knowledge has published postprandial RQ values in dogs^(^^22^^)^. Pouteau *et al.*^(^^22^^)^ also fed adult Beagle dogs a diet of similar composition to the HC diet. They reported comparable changes in postprandial RQ (0·91 (sem 0·01), 24 h average). However, the present study is the first to demonstrate changes in postprandial RQ in response to different dietary compositions. These data further validate the use of indirect calorimetry as a research tool to measure fasting and meal-induced responses in whole-body energy metabolism in dogs. Fasting RQ was greatest in dogs fed the HC^+MH^ diet, suggesting increased CHO oxidation relative to fat oxidation in fasting. The ability of MH to affect fasting RQ was unexpected, as previous reports have demonstrated that MH has transient effects on metabolism that are reversed within 1–6 h of administration^(^^11^^–^^15,^^19^^)^. The effect may be a result of chronic MH feeding. However, only one other study to our knowledge has examined the effects of chronic MH supplementation^(^^28^^)^, but those authors did not measure RQ. It is unclear whether changing resting RQ would have any biological significance. These results are somewhat surprising. As MH is purported to function as a competitive inhibitor of glucokinase, one may have expected MH to slow the rate of glucose utilisation, especially when faced with a high CHO (glucose) load. These results further demonstrate the need for additional research to evaluate the role of MH on glucose and fat oxidation in fasting and the postprandial period to see if they are repeatable.

In contrast to previous studies, MH did not affect serum glucose or insulin. Previous studies showed that high doses (>1·2 g/kg) of MH induced transient hyperglycaemia and hypoinsulinaemia in rats, dogs and human subjects^(^^14^^,^^16,^^29^^,^^30^^)^. However, these doses elicited responses well outside the range of normal metabolism, suggesting the dose was supraphysiological. Low dietary MH concentrations (2 mg/kg) have been reported to lower postprandial serum insulin concentrations, but not glucose, when fed to senior Labrador retrievers (average age 11·8 years) for 30 d^(^^28^^)^. The lack of a postprandial insulin response in the present study may be attributed to the fact that dogs were young and lean, which may have precluded any noticeable metabolic changes. It is worth noting that aside from Davenport *et al.*^(^^28^^)^, this is the only study to examine the effects of MH in the postprandial state and a small sample size was used. Owing to its ability to inhibit glucokinase in the small intestine, liver and pancreas, MH is quite likely to have differential effects on glycaemia in fasting and fed conditions.

Dogs fed the LC diet exhibited higher serum glucose than dogs consuming the HC diet. However, serum glucose values were within the normal range for healthy dogs. Serum insulin concentrations were not different between diets, suggesting that the LC diet induced slight insulin resistance. These findings may be attributed to the high fat relative to CHO content of the LC diet. High-fat diets have been shown to induce impairments in insulin-mediated glucose disposal in as quickly as 3–5 d in human subjects^(^^31^^–^^33^^)^. High fat, high fructose feeding for 6 to 12 weeks induced glucose intolerance in dogs^(^^34^^,^^35^^)^. However, to our knowledge no acute studies examining the effects of exclusively high fat (relative to CHO) have been performed in dogs. In order to fully understand the effects of feeding a diet high in fat relative to CHO on insulin sensitivity and glucose tolerance, more sensitive measures are needed partnered with a longer dietary adaptation period. In addition, measuring serum lipids, including TAG and NEFA, would provide insight into the metabolic adaptations that occur with high fat feeding. The minimal glucose response observed in dogs consuming the HC diet is consistent with other studies using dogs fed similar concentrations of dietary CHO^(^^34^^–^^36^^)^. In comparison with human subjects, dogs tend to have a blunted glucose response to a mixed meal which has been attributed to the prolonged absorptive phase (12–24 h) in dogs^(^^37^^)^. Furthermore, the HC diet had high inclusion of sorghum grain, which is considered to have a low glycaemic index in dogs due to its high dietary fibre content^(^^38^^)^.

In conclusion, MH has acute effects on postprandial EE and RQ. Specifically, MH increased fasting RQ, indicating an increase in CHO relative to fat oxidation. MH increased dietary induced thermogenesis in the high CHO relative to fat diet, supporting its use as an ER mimetic. Increasing dietary thermogenesis would be of particular benefit to overweight and obese animals, especially those consuming a high-CHO low-fat diet. Future research is warranted and needs to involve more sensitive measures of macronutrient oxidation to fully elucidate the mechanism of action of MH on whole-body metabolism in fasting and postprandial conditions. Irrespective of MH, the present study demonstrated that dogs adapt relative CHO to fat oxidation to diet composition. Despite this finding, dogs consuming a low-CHO high-fat diet had increased serum glucose.

## Supplementary Material

Supplementary Material Supplementary information supplied by authors.

## Supplementary material

To view supplementary material for this article, please visit http://dx.doi.org/10.1017/jns.2014.17
