# Supplementary material for: Mannoheptulose has differential effects on fasting and postprandial energy expenditure and respiratory quotient in adult Beagle dogs fed diets of different macronutrient contents
Source: J Nutr Sci. 2014 Aug 13;3:e17. doi: 10.1017/jns.2014.17 (PMC4153035; doi:10.1017/jns.2014.17)
Supplement: Supplementary Material — Supplementary information supplied by authors. [file S2048679014000172sup001.doc]

**Supplementary Fig. S1(a).** Post-prandial energy expenditure (EE, KJ/kg/d) as measured by indirect calorimetry in adult Beagle dogs (N = 8 in a cross-over design). Post-prandial measures were taken for 22.5 h after ingestion of a single test meal and supplements (low carbohydrate diet with placebo supplement (LC); mannoheptulose (8 mg/kg) containing supplement (LC^+MH^); high carbohydrate diet with placebo supplement (HC); mannoheptulose (8 mg/kg) containing supplement (HC^+MH^)) at time zero. Data were analyzed in time blocks of 0 to 4 h, 5 – 10 h, 11 – 17 h and 18 – 23 h. The main effect of diet was significant for the 5 – 10 h time block only (p = 0.02).

**Supplementary Fig. S1(b).** Post-prandial respiratory quotient (RQ) as measured by indirect calorimetry in adult Beagle dogs (N = 8 in a cross-over design). Post-prandial measures were taken for 22.5 h after ingestion of a single test meal and supplements (low carbohydrate diet with placebo supplement (LC); mannoheptulose (8 mg/kg) containing supplement (LC^+MH^); high carbohydrate diet with placebo supplement (HC); mannoheptulose (8 mg/kg) containing supplement (HC^+MH^)) at time zero. Data were analyzed in time blocks of 0 to 4 h, 5 – 10 h, 11 – 17 h and 18 – 23 h. The main effect of diet was significant for all time period (p < 0.01).
